# An Eye Tracking Study of Anti-Smoking Messages on Toxic Chemicals in Cigarettes

**DOI:** 10.3390/ijerph16224435

**Published:** 2019-11-12

**Authors:** Leah M. Ranney, Sarah D. Kowitt, Tara L. Queen, Kristen L. Jarman, Adam O. Goldstein

**Affiliations:** 1 Department of Family Medicine, UNC School of Medicine, University of North Carolina at Chapel Hill, 590 Manning Drive, CB# 7595, Chapel Hill, NC 27599, USA; Kowitt@unc.edu (S.D.K.); adam_goldstein@med.unc.edu (A.O.G.); 2 Lineberger Comprehensive Cancer Center, University of North Carolina at Chapel Hill, 101 Manning Drive, CB# 7295, Chapel Hill, NC 27599, USA; tlqueen@email.unc.edu (T.L.Q.); jkristen@email.unc.edu (K.L.J.)

**Keywords:** smoking, anti-smoking, chemicals, toxins, eye tracking, messages

## Abstract

The US Food and Drug Administration is tasked with communicating information to the public about the harmful chemicals in cigarette smoke. Our study used eye tracking method to test the effectiveness of messages about the harmful chemicals in cigarettes smoke among adult smokers. A sample size of 211 current cigarette smokers viewed four communication messages that included: Health effects of a chemical in cigarette smoke and an image depicting the health effect. The messages focused on arsenic, formaldehyde, uranium, and general health. Eye tracking recorded the length of time participants viewed the text and the image. After each message, the participants were asked about the messages’ effectiveness in changing attitudes towards smoking. We analyzed the data using multilevel modeling, and of the 211 smokers, 59.7% were female, 36.5% were Black, and 21.3% had a high school degree or less. Compared to the general message, the messages about formaldehyde and uranium were more discouraging to smoking (*p* < 0.05). Messages about formaldehyde were more believable and made participants want to quit more than the general messages. Increasing message dose was significantly associated with discouraging participants from smoking and made participants want to quit (*p* < 0.05). Our findings suggest that anti-smoking messages, containing chemical information, can successfully increase negative attitudes toward smoking cigarettes and potentially encourage quitting.

## 1. Introduction

Cigarette smoking is the leading cause of preventable death in the USA [1,2]. Smoke from cigarettes and other tobacco products, like cigars and pipes, contain at least 70 cancer-causing chemicals known as carcinogens [3]. Chemicals created from the process of curing tobacco leaves, that are added to enhance flavor and reduce harshness (i.e., additives), and that are created when the cigarette is burned cause many serious health-related illnesses, such as cancer, heart disease, stroke, lung diseases, diabetes, and chronic obstructive pulmonary disease [1,4,5]. Since the 2009 Family Smoking Prevention and Tobacco Control Act, the US Food and Drug Administration (FDA) has been charged with regulating the manufacture, distribution, and marketing of tobacco products [6]. Under their purview, the FDA is required to publicly disclose the ingredients (i.e., constituents) found in tobacco products. As part of this requirement, the FDA has established a list of 93 harmful and potentially harmful constituents (HPHC) in tobacco products and tobacco smoke, and the tobacco industry must report HPHC in their existing and newly marketed tobacco products [7].

An important question raised by the FDA’s new requirement is how best to communicate tobacco constituents and their risks to the public. The FDA’s initial effort to communicate the risk associated with chemicals in cigarettes smoke was with *The Real Cost* campaign, which was targeted towards youth [8]. Several of these anti-smoking advertisements highlight the harms associated with chemicals in cigarettes, like the “7000 chemicals” ad. While, the evaluation of *The Real Cost* reported the campaign was highly successful, no outcomes were reported specific to the constituent messages [9,10,11].

Recent research has shed some light on how to communicate the dangers of the chemicals and chemical constituents in tobacco products [12,13,14,15]. For instance, research found that many people are unaware that burning the cigarette is the primary source of harmful chemicals, and few believe the harmful chemicals come from tobacco before cigarettes are manufactured [13,16]. An early study examining the optimal methods to display tobacco constituent levels on tobacco packaging found adult perceived descriptive information as more comprehensible than numerically displayed information [17]. Recent studies testing messages with cigarette constituents information among adults showed that the messages increased the constituents’ level of knowledge, reinforced smoking related-health outcomes, and discouraged people from wanting to smoke [18,19].

While, research is building around cigarette smoke constituents, few studies have examined the effectiveness of communication campaigns where the constituents are part of the messaging. Even fewer have examined this using eye tracking technology [20,21], which provides measurable data on visual attention, a precursor to information processing that can supplement evidence from self-report methods [22]. Measuring visual attention to specific components of communication campaigns can improve our understanding of how people attend to, and are influenced by, the messages [23]. Our study uses eye tracking technology to experimentally test the effectiveness of anti-smoking messages, containing constituent information on print ads, among adult cigarette smokers. We hypothesized that, (1) constituent messages increase negative attitudes toward smoking cigarettes more than a non-constituent general health message about the consequences of smoking, and (2) looking at the message text and images longer would be associated with higher self-reported measures of the messages’ impact.

## 2. Methods

### 2.1. Participants

Recruitment of participants occurred between July and December 2016 from the research triangle area of North Carolina. Participants were between the ages of 18–65 years old and current smokers (i.e., had smoked more than 100 lifetime cigarettes and smoked every day or some days in the past 30 days). Participants were included in the study if they were current smokers. While, participants were asked about their use of other tobacco products, other tobacco use was not an eligibility criterion for this study. Participants who were not able to be eye tracked (e.g., did not have vision in both eyes) or who had participated in a tobacco study in the past 3 months were excluded. The study initially enrolled 230 participants. Of these participants, 5 were removed because the eye tracker was not able to calibrate to their eye movements, 4 did not complete the study protocol, and 10 were removed due to incomplete eye tracking data. The final sample was 211 participants. The University of North Carolina Institutional Review Board approved study procedures, and participants provided their written informed consent.

### 2.2. Procedures

This was a one-time eye tracking study. Before the study session began, the participants were randomly allocated to 1 of 4 conditions and remained in the same condition group throughout the study session. The 4 conditions were created as part of a 2 × 2 experimental design. The two experimental factors were (1) whether participants saw messages with the FDA logo and (2) whether participants saw messages that included text about the benefits of quitting smoking: Additional details about the study methodology can be found in Jarman L, Kowitt S, Queen, L. et al. [20].

**Figure 1:**
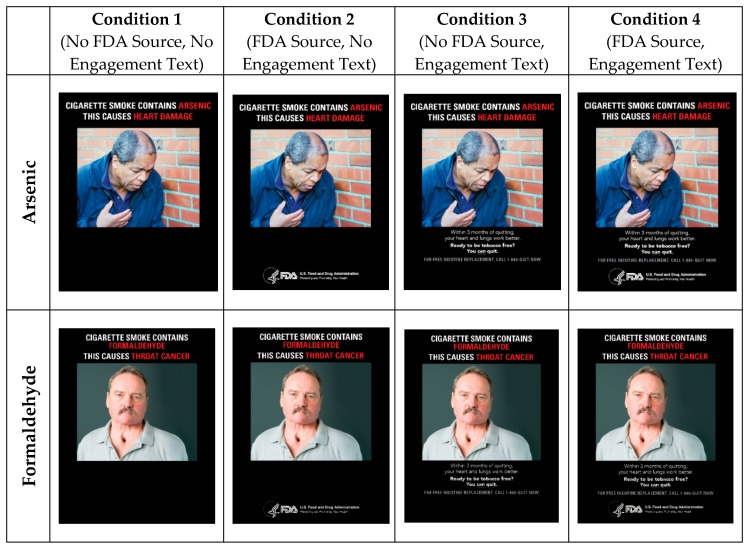

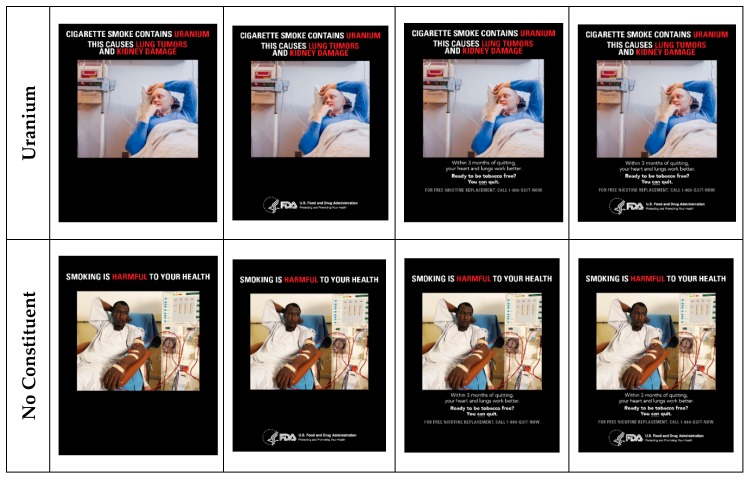
Stimuli by Condition for eye tracking experiment.

Within each of the 4 conditions, the participants saw 4 messages (Figure 1). The messages were formatted as hypothetical print campaigns about different constituents in cigarette smoke. All messages (regardless of condition), included (1) text about the health effects of a chemical constituent in cigarette smoking (e.g., cigarette smoke contains arsenic. This causes heart damage.) and (2) an image depicting the health effects of the chemical constituent. The 4 messages within each condition focused on the health effects of arsenic, formaldehyde, uranium, as well as a non-constituent general message about the health harms of smoking. Based on prior research assessing the understanding of 24 specific cigarette smoke constituents, the three constituents selected for the study messages were identified as most discouraging of smoking in a national probability sample of adults and adolescents [16]. Uranium, one of the top constituents from Brewer et al. contained two health effects of chemicals and was tested without altering this format. To develop the images and other relevant anti-smoking information, we used a series of focus groups and online experiments [24,25]. The images were intentionally selected to represent the message, and therefore, were not randomized with the message. At the beginning of the study session, participants’ eye movements were calibrated using a Tobii X2-60 eye tracker. Following calibration, participants then answered questions about their demographics and smoking behaviors. Participants were then shown the 4 constituent messages (i.e., in a counterbalanced order) and were allowed to view the messages for as long as they liked. After viewing each message, participants responded to questions about the persuasiveness and impact of these messages (see Measures). At the end of the eye tracking session, participants were asked additional questions about elements manipulated in the study (e.g., recall of source).

### 2.3. Measures

Our primary outcomes of interest were self-reported measures of the messages’ impact [26]. Our primary independent variables of interest were the constituent, attention to the image, attention to the constituent message text, and the effect of message dose.

#### 2.3.1. Outcome: Measures about Message Impact

After each message was presented, the participants were asked to report on how believable the message was (hereafter believability), how much the message made them want to quit smoking (want to quit), and the extent to which the message discouraged them from smoking (discouragement). Responses were scored on a scale of 1–9 where 1 = Strongly Disagree, 5 = Neutral, 9 = Strongly Agree.

#### 2.3.2. Message-Related Variables

In order to determine which constituent messages were more impactful, we created a categorical variable for the message (i.e., arsenic, formaldehyde, uranium, or general, with general being the referent group). Eye tracking variables of interest included the total length of time participants attended to the image (image dwell time) and text (text dwell time). Dwell time is a measure of visual attention that, by our definition, included both fixations within an area of interest, as well as saccades between those fixations [27]. Since dwell time for the entire print ad was highly correlated with both text and image dwell time, we analyzed the image dwell time and text dwell time separately. To determine whether there was a cumulative impact of messages over the course of the study, we created a continuous variable that captured message dose. For example, the first message that a participant viewed was coded as 0 previous messages, the second was coded as 1 previous messages, etc.

#### 2.3.3. Control Variables: Demographics and Condition

Demographic measures, collected at the beginning of the study session, included: Age, sex at birth, sexual identity, ethnicity, race, education, income, and nicotine dependence. Nicotine dependence was assessed using the Fagerström Test for Nicotine Dependence (FTND), and responses were scored on a scale of 0–10 [28]. We controlled for these demographic measures in all models. In addition, we also controlled for the condition to which participants were assigned.

### 2.4. Data Analysis

#### 2.4.1. Modeling Strategy

After examining the data descriptively, we analyzed data using 3 multilevel models that corresponded with each of the 3 outcomes. We used a multilevel modeling approach to account for multiple observations (4 messages) within participants over the course of the study. In order to parse between- versus within-person variance in dwell times, we grand and group mean-centered these variables. In grand mean-centering dwell time, we calculated the deviation of each participant’s score from the overall mean of each variable. In group mean centering, we calculated the deviation of each observation from the mean for the participant’s cluster (participants in this case). For clarity, we labeled all observation-level, stimuli specific variables (dwell time, believability, etc.) as “Level 1” variables and labeled all participant-level variables (demographics, nicotine dependence, etc.) as “Level 2” variables.

#### 2.4.2. Multilevel Models

After centering, we examined the associations among the variables and outcomes, adjusting for control variables. We set critical α = 0.05 and used 2-tailed statistical tests. For all analyses, we used SAS version 9.4 procedures (SAS Inc., Cary, NC, USA). For the multilevel models, we used the PROC MIXED procedure. Since each participant saw 4 different messages and there were 211 participants, there were 844 unique observations (211 × 4).

## 3. Results

### 3.1. Participant Characteristics

Of the 211 participants, the majority were female (59.7%) and White (58.3%), and a substantial proportion of participants identified as African American (36.5%) (Table 1). The average age of participants was 36.2 years (standard deviation, SD: 12.4), and the average nicotine dependence score was 4.13 (SD: 2.59).

### 3.2. Descriptive Statistics

On average, the formaldehyde message scored the highest on all 3 outcomes. Specifically, the formaldehyde message was rated as more believable, made participants want to quit more, and discouraged them the most from smoking (Table 2). Interestingly, the arsenic message scored the lowest on all 3 outcomes.

Participants spent the most time looking at the image for the general message (4.39 s, SD: 4.61) and the least amount of time looking at the image for the uranium message (2.67 s, SD: 2.89). In contrast, we found that participants spent the most amount of time looking at the uranium message text (3.33 s, SD: 3.54) and the least amount of time looking at the general message text (0.41 s, SD: 0.65).

### 3.3. Multilevel Models

When we modeled believability as the outcome, we found that the arsenic message (B = −0.53, *p* < 0.001) and the uranium message (B = −0.42, *p* < 0.001) were less believable than the general message (Table 3). The formaldehyde message (B = 0.30, *p* < 0.02), was more believable than the general message. There were no effects of image or text dwell time on believability, nor was there an effect of message dose.

When we modeled wanting to quit as the outcome, we found that the formaldehyde message (B = 0.97, *p* < 0.001) made them want to quit more than the general message. There were no effects of image or text dwell time on the messages’ ability to help participants to quit. However, as message dose increased, they reported that the messages made them want to quit smoking more (B = 0.13, *p* < 0.001).

Finally, when we modeled discouragement as the outcome, we found that the formaldehyde message (B = 1.05, *p* < 0.001) and the uranium message (B = 0.43, *p* = 0.01) discouraged participants from smoking more than the general message. There were no effects of image or text dwell time on the messages’ ability to help participants to quit. However, as participants viewed more messages, they reported that the messages discouraged them more from smoking (B = 0.13, *p* = 0.004).

## 4. Discussion

This is one of the first studies using eye tracking technology to test the impact of cigarette smoke constituent messages among adult smokers. Using the results from previous research, we created four anti-smoking print ads by pairing text messages about constituents with images (three ads featured constituents and one featured a non-constituent, general health message). In addition to survey items, eye tracking technology was used to measure participants’ dwell time on the constituent text message and images contained within the ad. Our study indicates that constituent focused anti-smoking print ads performed differently than general anti-smoking print ads about the health harms of smoking among adult smokers, and that some constituent ads were more believable and influential than others. Specifically, the formaldehyde constituent ad was more believable, made smokers want to quit more, and discouraged smoking more, when compared to the general health ad (i.e., non-constituent ad). While the uranium constituent ad was also reported to be more discouraging and made participants want to quit more than general health ads (non-constituent), the arsenic and the uranium constituent ads were less believable than the general health ad. Research on constituent messages provides some insight into the nuances that may differentiate constituent messages from each other [12,13,15,16,29].

Research on cigarette smoke constituents suggest that, while knowledge of cigarette smoke constituents is low, most people have heard of nicotine, carbon monoxide, ammonia, arsenic, benzene, cadmium, and formaldehyde [13,15,16]. Brewer et al. found that the more recognizable constituents—ammonia, arsenic, formaldehyde, hydrogen cyanide, lead, and uranium—were most likely to discourage respondents from wanting to smoke [16]. Our study applied the findings from Brewer et al. and used the most familiar and effective constituents to produce the constituent print ads. An additional finding from Brewer et al. was that longer constituent names were more discouraging than shorter names. This may explain why the arsenic constituent ad scored the lowest on all of the outcomes (believability, wanting, discouragement) and the formaldehyde constituent ad was the most impactful constituent ad on all three outcomes. The image for the formaldehyde constituent ad, a man with a laryngectomy, was also more aversive or attention-grabbing than the other images, and may have contributed to higher message believability and greater desire to quit. Importantly, message believability has been shown to be associated with the intention to engage in smoking cessation behaviors [30].

Tobacco control mass media campaigns can promote quitting and reduce adult smoking prevalence when factors, such as audience reach, intensity, duration, and message type are successful [31]. Media campaigns have also linked cigarette smoking to a range of diseases with research suggesting that cancer-related anti-smoking messages are 1.5 times more believable than chronic disease messages [14]. Additionally, constituent messages, focused on cancer and health effects related to the lungs, mouth, and heart generally led to higher levels of smoking discouragement [15]. Our research supports these findings. Participants rated the formaldehyde and uranium constituent ads (which focused on cancer, and health effects related to the lungs, respectively) as more discouraging than the general message. Interestingly, the formaldehyde constituent ad had the only message containing the word cancer in the related-health effect, and it was the only constituent ad reported to be more believable than the non-constituent ad with the general message. Research suggests that pairing known information (i.e., smoking causes cancer) with new information (i.e., formaldehyde cause cancer) influences message processing and beliefs [14,32]. This may be an important principle for future constituent communication campaigns.

Notably, as participants in our study viewed more ads, they reported that the ad messages were more effective. This finding is consistent with evidence supporting the effect of increased message exposure on promoting smoking cessation [32,33]. Health message campaigns are often evaluated on the number of impressions, with the expectation that more campaign exposure may lead to increased effectiveness of the campaign [34,35]. Our findings provide initial support that constituent ads can successfully discourage smoking and potentially made smokers want to quit more with repeated message exposure.

Because previous research suggests that pictorial messages are more effective than text-only messages [36,37], our study paired each of the constituent messages with an image depicting the health effect to increasing ad effectiveness. As an indicator of visual attention, our study used eye tracking technology to measure participant dwell time on the constituent text and image for each constituent print ad. Eye tracking allows researchers to move beyond self-reported outcomes and observe participants’ actual visual attention [23]. Not surprisingly, participants spent more time on the longer text message on the uranium constituent ad. This ad contained two health effects opposed to the other constituent ad, with only one health effect and the non-constituent ad message. The least amount of time was spent on the text for the non-constituent ad, potentially due to the brevity of the message. Interestingly, the reverse was true for dwell time on the image; the image for the non-constituent ad had the longest dwell time, while the uranium constituent ad image had the shortest dwell time.

We found no effects from increasing image or text dwell time on any of the outcomes. The majority of participants did indeed look at the image and message text, and the research indicates that information acquisition from a visual stimuli can occur in a fraction of a second (i.e., 0.1 s) [38,39,40,41]. The participants in our study spent, on average, approximately 3–4 s on the image, and 1–3 s on the text for each message, which indicates that they spent a significant amount of time attending to the information and potentially processing it. Participants may have more carefully viewed each ad because research staff was present during the experiment, and because of the incentive offered for the study. However, participants were allowed to proceed with the experiment at their own pace. Hence, the absence of text and image dwell time effects might be due to study protocol and conscious participant behavior, resulting in the majority of participants adequately processing the message.

Overall our findings suggest that an anti-smoking campaign that uses constituent information that is familiar to the public and features the negative health effect of the constituent can successfully discourage smoking and persuade adult smokers to want to quit. Importantly, repeated ad exposure increased the impact of the constituent ads, indicating that multiple exposure to ads via a mass media campaign can increase persuasion over time.

### Limitations

Our study is not without limitations. First, this was a one-time study in a laboratory setting where participants had to view the constituent messages. The results, therefore, may not be generalized to other settings. Relatedly, participants were recruited from one geographic area, which also may have limited the generalizability to other areas. While, there is a strong link between eye tracking fixations and attention, it is possible that, while, a person’s visual gaze is within an AOI, they are not actually processing that information. It is possible that when looking within our defined AOIs, that participants were processing peripheral information or thinking about something else entirely [42,43,44]. Therefore, we cannot determine whether participants were processing information associated with those areas of interest. The intention of the study was to assess whether messages as a whole increased negative attitudes and intentions to quit smoking rather than to choose the best image or message. Therefore, we intentionally did not randomize images with the messages and cannot disentangle the effects of the message text and image on outcomes. Finally, we assessed self-reported outcomes after each ad exposure. However, there were a number of factors that could have influenced these outcomes, including the message text and the message image.

## 5. Conclusions

Our study findings have significant implications for the FDA and their requirement to disclose cigarette smoke constituent information to the public in an understandable and not misleading manner. Further, our study anti-smoking constituent campaign ads are consistent with established standards for effective tobacco communication [45]. Formaldehyde, a familiar constituent, and throat cancer, a familiar health effect, produced the most believable and influential constituent ad message for adult smokers in this study. Educational campaigns that communicate well-designed constituent ads, with corresponding images, may help reduce smoking among adults.

## Figures and Tables

**Table 1:** Participant characteristics, *n* = 211.

Variable	*N* (%) or Mean (SD)
Age, years (mean, SD)	36.23 (12.43)
Sex at Birth	
Female	126 (59.72%)
Male	85 (40.28%)
Sexual Identity	
Straight or Heterosexual	174 (82.46%)
Gay, Lesbian, Bisexual, Other	37 (17.54%)
Ethnicity	
Not Latino/Hispanic	200 (94.79%)
Latino/Hispanic	11 (5.21%)
Race	
White	123 (58.29%)
Black or African American	77 (36.49%)
Other	11 (5.21%)
Education	
Greater than high school	166 (78.67%)
High school graduate or less	45 (21.33%)
Income	
Below $25,000	87 (41.23%)
$25,000–$49,999	84 (39.81%)
Greater than $50,000	40 (18.96%)
Nicotine dependence score (mean, SD) (range = 0–10)	4.13 (2.59)

**Table 2:** Descriptive statistics for self-reported outcomes by message constituent, *n* = 211.

Message Constituent	Dwell Time (Seconds), Mean (SD)			Outcome, Mean (SD)		
	Image	Text	Full Ad	Believability	Want to Quit	Discouragement
Arsenic	2.74 (3.17)	1.80 (1.80)	9.88 (6.96)	6.07 (2.07)	4.75 (2.38)	4.72 (2.36)
Formaldehyde	3.32 (3.89)	1.97 (1.92)	9.00 (6.43)	6.90 (1.39)	5.91 (2.01)	5.73 (2.24)
Uranium	2.67 (2.89)	3.33 (3.54)	9.50 (6.77)	6.16 (1.89)	5.23 (2.26)	5.08 (2.39)
General	4.39 (4.61)	0.41 (0.65)	9.66 (8.38)	6.61 (1.64)	4.87 (2.17)	4.73 (2.29)

Note: mean dwell time for image was 3.28 (SD: 3.76), mean dwell time for text was 1.88 (SD: 2.45), and mean dwell time for the full ad was 9.51 (SD: 7.17).

**Table 3:** Multivariable repeated measures outcomes, *n* = 211 participants and *n* = 844 observations.

Message Constituent	Outcome		
	Believability B (*p*-Value)	Want to Quit B (*p*-Value)	Discouragement B (*p*-Value)
Arsenic (ref. general)	**−0.53 (*p* < 0.001)**	−0.19 (*p* = 0.15)	0.02 (*p* = 0.91)
Formaldehyde (ref. general)	**0.30 (*p* = 0.02)**	**0.97 (*p* < 0.001)**	**1.05 (*p* < 0.001)**
Uranium (ref. general)	**−0.42 (*p* < 0.001)**	0.21 (*p* = 0.17)	**0.43 (*p* = 0.01)**
Level 1 image dwell time	0.00 (*p* = 0.81)	0.00 (*p* = 0.93)	−0.02 (*p* = 0.30)
Level 2 image dwell time	0.04 (*p* = 0.36)	0.05 (*p* = 0.37)	0.08 (*p* = 0.16)
Level 1 text dwell time	−0.01 (*p* = 0.82)	0.05 (*p* = 0.07)	−0.04 (*p* = 0.22)
Level 2 text dwell time	0.01 (*p* = 0.89)	0.07 (*p* = 0.45)	0.00 (*p* = 0.99)
Message dose	−0.05 (*p* = 0.18)	**0.13 (*p* < 0.001)**	**0.13 (*p* = 0.004)**

Analyses control for sex at birth, age, sexual identity, race, ethnicity, income, education, nicotine dependence score, and condition. Boldface indicates significance at *p* < 0.05.
